# Hepatitis-A Infection-Induced Secondary Antiphospholipid Syndrome With Neuro-ophthalmological Manifestations

**DOI:** 10.7759/cureus.20603

**Published:** 2021-12-22

**Authors:** Nora D Al-Dohayan, Fatma Al-Batniji, Hammam A Alotaibi, Abdulaziz T Elbaage

**Affiliations:** 1 Pediatric Ophthalmology, Prince Sultan Military Medical City, Riyadh, SAU; 2 Pediatric Department, Prince Sultan Military Medical City, Riyadh, SAU; 3 Ophthalmology, Dhahran Eye Specialist Hospital, Dhahran, SAU; 4 Research Center, Prince Sultan Military Medical City, Riyadh, SAU; 5 Department of Medicine, Presbyterian St. Luke's Medical Center, Denver, USA

**Keywords:** anticoagulation therapy, pediatrics ophthalmology, clinical hematology, antiphospholipid syndrome, hepatitis a

## Abstract

Antiphospholipid syndrome (APS) is a multisystem autoimmune disorder that can affect children and adults alike, with a similar spectrum of thrombotic events, predominately deep vein thrombosis and stroke. It is characterized by recurrent arterial or venous thrombosis and recurrent fetal loss with the presence of antiphospholipid antibodies (aPL) like antibodies to beta-2-glycoprotein I (B-2-GPI) and anticardiolipin (aCL). The disease could be classified into primary APS in the absence of an underlying disease or secondary APS occurring secondary to autoimmune diseases, infections, malignancies, and sometimes medication use. In the absence of clinical manifestations of APS, transient non-thrombogenic antiphospholipid antibodies are seen more commonly in children, predominantly after childhood infections. Cases with clinical manifestations of APS associated with different types of infections have been reported in the literature to keep track of potential triggering causes and take measures to prevent or treat the disease manifestations. This case documents the case of hepatitis-A as a triggering viral infection, causing secondary APS in a child.

## Introduction

Antiphospholipid syndrome (APS) is an autoimmune disease that impacts both children and adults alike with the same diverse spectrum of thrombotic events such as deep vein thrombosis and stroke [1]. It is characterized by a recurrent artery or venous thrombosis leading to manifestations such as stroke or deep vein thrombosis. It can also lead to fetal loss in pregnancy where the dominant feature at the time of the loss is the presence of antiphospholipid antibodies (aPL), especially antibodies to beta-2-glycoprotein-I (B-2-GPI) and anticardiolipin (aCL) [2].

The disease can be classified as primary or secondary depending on the cause. Primary disease is characterized by the absence of any identifiable underlying causes at the time of diagnosis, while the secondary disease is characterized by an identifiable cause such as autoimmunity, infections, malignancies, or a precipitating factor like using certain medications [3,4].

In children, transient non-thrombogenic antiphospholipid antibodies are observed more commonly in the absence of other clinical manifestations of APS. They are observed most commonly after severe infections during early childhood [1]. Cases of different clinical manifestations of APS syndrome during childhood are documented in the literature and correlated with the potential infectious cause precipitating the disease.

This case report documents the neuro-ophthalmological manifestations of hepatitis-A infection inducing antiphospholipid syndrome in a child, a manifestation that is exceedingly rare in the literature.

## Case presentation

An 11-year-old boy was referred from a peripheral hospital to the neurosurgery department as a case of left-sided intracranial hemorrhage for further management. His complaint started one month after his presentation with a history of frequent attacks of frontal headache not relieved by analgesia and not associated with other symptoms, followed by a high-grade fever of 39-40 °C for two weeks, treated with antibiotics and antipyretics. His condition progressed to projectile vomiting and lethargy with diplopia starting two days before presentation. There was no history of trauma or convulsions. He was admitted to the local hospital and started on antihypertensive therapy due to high blood pressure and also received antiepileptic therapy. A computer tomography (CT) scan of the brain was done, which showed left occipital intracranial hemorrhage. He was referred to our hospital for further management. The patient has a past history of hepatitis-A infection and jaundice one month before the presentation (proven by a serology lab test), which was treated conservatively. His vaccinations were up to date, and his development is normal for his age. There was no family history of any similar illness, blood diseases, consanguinity, or maternal history of abortion or neonatal death.

A general inspection showed that he was ill-looking, depressed, not distressed, fully conscious and alert, overweight for his age, not pale or jaundiced, and had no lymphadenopathy. There is some bruising on the lower limbs. The temperature was 36 °C, the respiratory rate was 25/min, the heart rate was 85/min, the blood pressure was 119/78, the oxygen saturation was 98%, the weight was 54 kg, and the height was 145 cm. The chest was clear. The cardiovascular examination was normal. The abdomen was soft with no organomegaly. The central nervous system examination showed no facial asymmetry, bilateral sixth cranial nerve palsy, and other cranial nerves were intact. Power, gait, motor and sensory functions were normal. He was referred to the ophthalmologist because of the horizontal diplopia, which was getting worse towards the right and left gazes.

**Figure 1:**
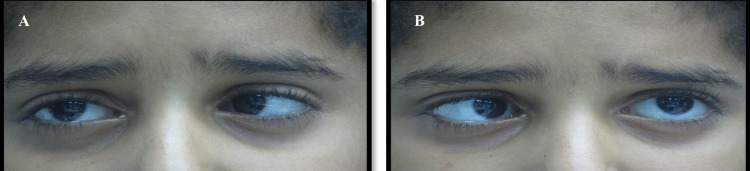
Limitation of abduction in the (A) right eye. (B) Left eye with failure to cross the midline on abduction bilaterally.

An ophthalmological examination showed that the best-corrected visual acuity (BCVA) was 6/6 in both eyes. The pupil is round, regular, and reacts with a negative afferent pupillary defect. Ocular motility shows limitations in the abduction of the right and left eyes; they could not abduct beyond the midline in both eyes as shown in Figure 1.

**Figure 2:**
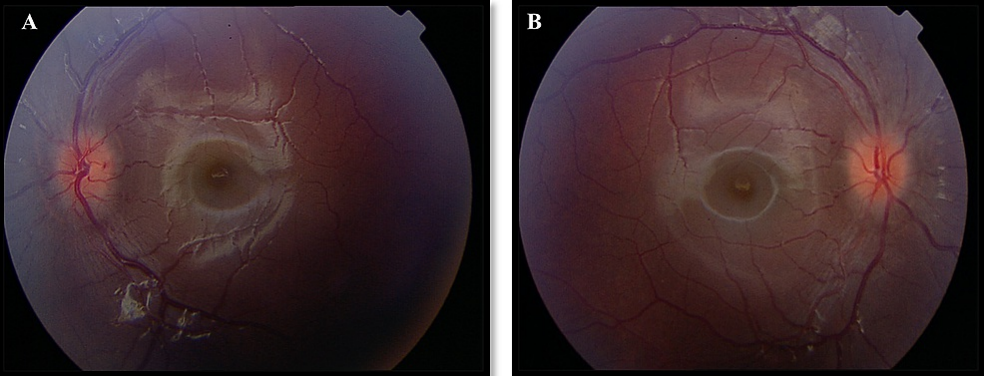
Patient's fundus photos showing (A) left eye optic disc with blurred margins and splinter hemorrhage and (B) right optic disc with blurred margins and tortuous retinal vasculature.

The deviation was 35 prism diopters of esotropia in the primary gaze. Slit-lamp biomicroscopy shows a normal anterior segment. Fundus examination of both eyes revealed blurry margins of the optic disc (Figure 2A, 2B) with splinter hemorrhage of the left optic disc (Figure 2B), mild tortuous blood vessels, and no venous pulsation. The visual field was normal in both eyes. An ophthalmic diagnosis of bilateral sixth nerve palsy with bilateral disc swelling was made. Blood investigations, CT-scan, lumbar puncture, abdominal ultrasound, and MRI were done.

 Blood investigations are shown in Table 1.

**Table 1:** Blood tests showing complete blood count and coagulation profile at baseline and after three weeks. RDW: red blood cell distribution width, WBC: white blood cell count, MCV: mean corpuscular volume, PT: prothrombin time, PTT: partial thromboplastin time, INR: international normalized ratio.

Test	Findings
WBC	9.2 × 10^9^/L
MCV	63 × 10^−15^/L
Platelets	65 × 10^9^/L
Hemoglobin	11.20 g/dl
Hemoglobin electrophoresis	HbA 97%, HbA₂ 2.6%, HbF 0.4% (picture of Alpha Thalassemia trait)
RDW	14.8%
Reticulocytes	1.8%,
Blood group	A−, positive direct Coombs test
Coagulation profile on admission	
PT	16.9
PTT	48
INR	1.8
After three weeks	
PT	10.3
PTT	40
INR	1.1

Serum levels of lupus anticoagulant, protein C and protein S, antithrombin and factor V Leiden and C3, C4 were normal. Septic screens for syphilis, human immunodeficiency virus (HIV), hepatitis-B virus (HBV), hepatitis C virus (HCV), and cytomegalovirus (CMV) were negative. The Epstein-Barr virus (EBV) IgM screen was negative and the EBV IgG screen was positive. An assay of antiphospholipid antibodies is shown in Table 2.

**Table 2:** Patient's measured antiphospholipid antibodies on admission and after nine months. GPL: IgG phospholipid units, IgG: immunoglobulin G, IgM: immunoglobulin M, IU/ml: international units per milliliter, MPL: IgM phospholipid units. Normal reference range in brackets.

Antiphospholipid antibodies	On admission	After nine months
Anticardiolpin IgG	100 GPL (0–23)	19 GPL (0–23)
Anticardiolpin IgM	56 MPL (0–11)	6 MPL (0–11)
Antiphospholipid IgG	70 GPL (0–15)	25 GPL (0–15)
Antiphospholipid IgM	95 MPL (0–21)	11 MPL (0–21)
B-2-GPI IgG	34 IU/ml (0–20)	3 IU/ml (0–20)
B-2-GPI IgM	11 IU/ml (0–20)	1 IU/ml (0–20)

A computer tomography scan (CT-scan) of the brain shows dural venous sinus thrombosis involving the superior sagittal sinus, inferior sagittal sinus, and transverse sinus with no evidence of raised intracranial pressure. A lumbar puncture was done with an opening pressure of 36 cm and closing pressure of 25 cm of water. Biochemistry and microbiology studies for cerebrospinal fluid (CSF) were normal.

**Figure 3:**
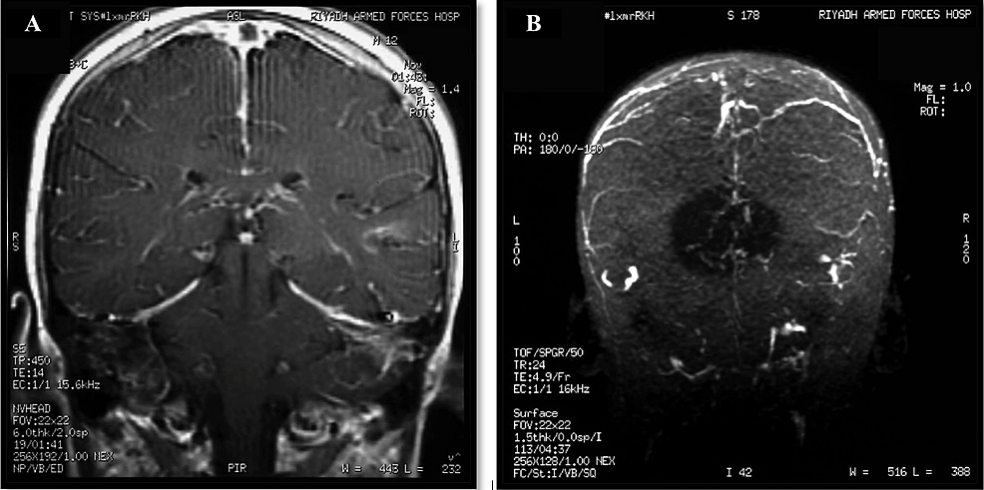
Patient's MRI and MRV showing: (A) coronal T1 post-contrast venogram of the brain demonstrate non-opacification of the superior sagittal sinus with empty delta sign as well as the transverse sinuses. (B) Single MIP image from a 3D SPGR post-contrast demonstrating non-visualisation of the superior sagittal sinus, inferior sagittal, and transverse sinuses. MRI: magnetic resonance imaging, MRV: magnetic resonance venography, MIP: maximum intensity projection, 3D SPGR: three-dimensional spoiled gradient recalled echo, T1: longitudinal relaxation time.

Magnetic resonance imaging (MRI) and magnetic resonance venogram (MRV) of the brain shows extensive thrombosis of the superior sagittal sinus, inferior sagittal sinus, transverse sinus, and an area of venous infarction in the left temporal lobe (Figure 3A, 3B).

The cardiac evaluation showed a small perforated foramen ovale. The chest X-ray and abdominal ultrasound were normal. A diagnosis of antiphospholipid syndrome was made. Treatment was started on low molecular weight heparin at 1 mg/kg every 12 hours. He was continued on the same dose with dose adjustment by monitoring antifactor Xa level at a therapeutic level of 0.5-1.00 I/U.

**Figure 4:**
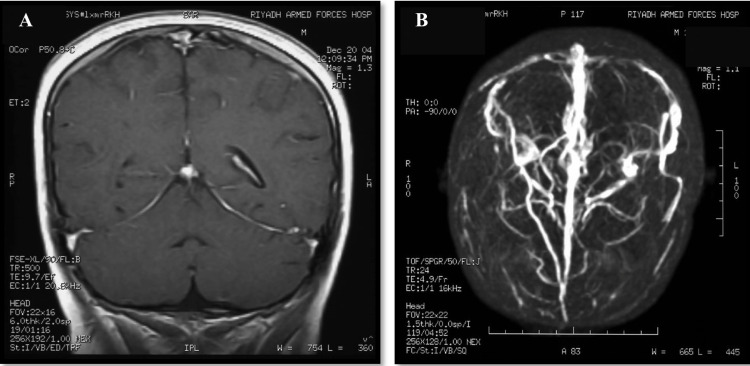
Follow-up imaging showing (A) coronal T1 MRV with good opacification of the venous sinuses and (B) follow-up post-contrast 3D MIP demonstrating re-canalization of the dural venous sinuses. MRV: magnetic resonance venogram, 3D MIP: 3-dimensional Maximum intensity projection, T1: longitudinal relaxation time.

After six weeks, the diplopia improved. Repeated blood investigations showed a gradual decrease of aCL, aPL, and B-2-GPI, but a positive dilute Russell's viper venom time (DRVVT). Repeated MRI and MRV (Figure 4A, 4B) showed recanalization of the thrombosed sinuses and a significant reduction in thrombus size. As shown after nine months (Table 2), antiphospholipid antibodies, anticardiolipin antibodies, and B-2-GPI were decreasing to reach normal levels.

## Discussion

Evidence is currently mounting that APS is caused by a mechanism of two parts: the first is the interaction of high levels of aPL antibodies with coagulation elements, causing dysfunction of the vascular endothelium. The second is the presence of a precipitating factor, including infection, use of medication, or a genetic element [4,5].

The presented case demonstrates a phenomenon documented in the literature, namely, the development of APS post infection in pediatric patients. This association between APS and infection has been studied and reported in the literature where the potential association varies from simply elevated aPL antibodies without systemic manifestations of symptomatic APS to catastrophic APS complications with death as a consequence [6].

A systematic review was conducted that included case reports describing the association of APS with different viral infections. Out of 2,278 cases, 200 were documented to develop APS post infection. These patients were categorized into two different groups. The first group included patients with clinical and immunological findings that fulfilled the APS criteria associated with a confirmed history of infection preceding the occurrence of APS. Various types of bacterial, viral, parasitic, and fungal infections were reported as triggering factors of APS. Most commonly reported infections include HIV, HCV, and mycoplasma pneumonia. The second group included patients who did not fulfil the criteria for the diagnosis of APS but had transient positive aPL antibodies existing during and after infection. Almost 60% of the second group developed thromboembolic attacks during the infection, with or without other coagulation disorders. The correlation between the development of antibodies and thrombosis has not been clearly established. This thrombotic event could be triggered by the infection itself, or a separate co-existent hypercoagulable state [7].

Moreover, a new case report documents similar findings where a viral infection precipitated APS in a young child who was successfully treated with anticoagulation therapy. This case is similar to the case presented herein except that the viral entity precipitating APS syndrome is HIV [8].

## Conclusions

In this case report, the only infection that preceded the patient’s illness was a hepatitis-A infection, which could be the trigger that precipitated APS. The occurrence of thrombosis and infection indicates a causative association between them rather than the coincidental occurrence of two separate unrelated events. The progressive decrease of aCL, aPL antibodies, and plasma B2GP1 reaching normal values within nine months after management with anticoagulant demonstrates a transient episode of APS. According to these findings, a diagnosis of secondary APS triggered by hepatitis-A infection was concluded.

The value of this case report stems from APS being the number one cause of thrombotic events in the pediatric patient group. This is why approaching the disease carefully and documenting the potential precipitating factors is of extreme value to clinicians. Hepatitis-A, as documented by this case report, is shown to be among the precipitating factors and need not be ignored when an acute event is seen either by the ER or by the pediatric services. Being aware of this potential serious delayed complication can save a life. To the best of our knowledge, this is the first reported case with acute hepatitis-A infection who developed an episode of APS where he was treated successfully with anticoagulation therapy and resolved within nine months.
